# Correlations among age, sex, axial length, and subfoveal choroidal thickness in the choriocapillaris structure analyzed using multiple en face image averaging optical coherence tomography angiography

**DOI:** 10.1371/journal.pone.0259880

**Published:** 2021-11-15

**Authors:** Ai Ichioka, Sotaro Ooto, Akihito Uji, Saki Manabe, Chieko Shiragami, Akitaka Tsujikawa

**Affiliations:** 1 Department of Ophthalmology and Visual Sciences, Kyoto University Graduate School of Medicine, Kyoto, Japan; 2 Department of Ophthalmology, Kagawa University Faculty of Medicine, Kagawa, Japan; Massachusetts Eye & Ear Infirmary, Harvard Medical School, UNITED STATES

## Abstract

**Purpose:**

To analyze the structure of the choriocapillaris in healthy eyes by using averaged en face images acquired using swept source optical coherence tomography angiography and to examine the changes in the macular profile in relation to age, sex, axial length, and choroidal thickness.

**Methods:**

This prospective, cross-sectional study included 81 eyes of 81 subjects without ophthalmologic or systemic diseases who underwent a full ophthalmologic examination, including 3 × 3-mm macular optical coherence tomography angiography. Four to nine choriocapillaris en face images were registered and averaged. The averaged images were then binarized and analyzed.

**Results:**

The averaged choriocapillaris images showed a continuous capillary meshwork, whereas the unaveraged images had a granular appearance. The mean total area and size of flow voids were 0.99 ± 0.20 mm^2^ and 567.8 ± 201.5 μm^2^, respectively, and these values correlated positively with age (p = 0.002, R = 0.336 and p = 0.026, R = 0.247, respectively). Age-related gains in the mean total area and flow void size were 4.20 × 10^−3^ mm^2^ and 3.07 μm^2^ per year, respectively. However, the mean total area and flow void size had no significant correlation with axial length, subfoveal choroidal thickness, or sex.

**Conclusions:**

Multiple averaged en face swept source optical coherence tomography angiography is more effective than a single optical coherence tomography angiography scan for better visualizing the choriocapillaris. The total area and size of flow voids within a 3 × 3-mm macular area positively correlated with age. This technique can be useful for investigating the changes arising in macular diseases.

## Introduction

The choroidal vasculature mainly supplies nutrients to the outer retinal layers and retinal pigment epithelium (RPE). The choriocapillaris—an anastomosing capillary layer of the choroid—lies posterior to Bruch’s membrane [1–4]. Both clinical and histopathological studies [5–9] have suggested that choroidal circulation is associated with retinal disorders such as age-related macular degeneration and diabetic retinopathy. Thus, the visualization of the choroidal vasculature and blood flow is of great importance in such disorders.

However, imaging the choriocapillaris in vivo has long been challenging [10]. Capillaries in the choriocapillaris are fenestrated [11], and hence, imaging the choriocapillaris by using dye-based angiography, such as fluorescein and indocyanine green angiography, is difficult because of the potential for dye leakage [12]. Moreover, the limited depth and lateral resolution offered by such imaging modalities makes it challenging to visualize the choriocapillaris network.

Optical coherence tomography angiography (OCTA) enables detailed visualization of the retinal and choroidal microvasculature through motion contrast of blood flow [13–15]. It is a noninvasive imaging technique that does not necessitate dye injection and has no limitations caused by dye leakage. Moreover, owing to its high depth resolution, the choriocapillaris layer can be selectively visualized.

Structural analysis of the choriocapillaris by using single OCTA images has been performed in healthy eyes [16], as well as in those with age-related macular degeneration [17, 18], diabetic retinopathy [19], placoid chorioretinitis [20], and myopia [21]. However, owing to the complex microstructure of the choriocapillaris, imaging it using current OCTA technology remains challenging.

Recent studies reported that averaging multiple en face OCTA images improved the image quality of the retinal vasculature and choriocapillaris by reducing speckle noise and compensating for the signals missing in a single image; moreover, this technique had a significant effect on quantitative measurements [22–24]. Owing to the use of a longer wavelength, swept-source OCT (SS-OCT) with a high scan rate offers higher penetration than does standard spectral-domain OCT (SD-OCT); furthermore, it helps acquire high-contrast images of the choroid. Averaging of multiple en face SS-OCTA images enables the visualization of the meshwork structure of the choriocapillaris in vivo, and is hence suitable for quantitatively assessing the choriocapillaris microstructure. The purpose of this study was to analyze the structure of the choriocapillaris in a large group of healthy eyes by using averaged en face SS-OCTA images, and to report the changes in the macular profile in relation to factors such as age, sex, axial length, and subfoveal choroidal thickness. Our findings revealed that the mean total area and flow void size correlated with age but not with axial length, subfoveal choroidal thickness, or sex.

## Methods

This study was approved by the Institutional Review Board and Ethics Committee of Kyoto University Graduate School of Medicine. It was carried out in accordance with the ethical standards stated in the Declaration of Helsinki. Written informed consent was obtained from all the participating subjects.

### Subjects

In this prospective, cross-sectional study, data were collected at Kyoto University (Kyoto, Japan) and Kagawa University (Kagawa, Japan) from October 2017 to April 2019. All the included subjects were Japanese, and they did not have any ophthalmologic disease. They underwent examinations, including autorefractometry/keratometry, best-corrected visual acuity measurement using a 5-m Landolt chart, axial length measurement using an IOL Master (Carl Zeiss Meditec, Dublin, CA), slit-lamp examinations, intraocular pressure measurements, and fundoscopy or color fundus photography. The exclusion criteria were as follows: best-corrected visual acuity worse than 20/25; refractive error > +5.0 or < -6.0 diopters; axial length > 27 mm; and intraocular pressure ≥ 22 mmHg. Ophthalmologists examined the fundoscopy or color fundus photographs to diagnose whether an eye showed any evidence of ophthalmologic abnormalities, such as optic neuropathy or retinal disease. Eyes determined to have ophthalmologic abnormalities were excluded.

Subjects with systemic diseases like diabetes mellitus or those with poorly controlled hypertension were also excluded. Although both the eyes of the subjects were recruited for the study, only one eye of each subject was randomly chosen for inclusion.

### Image acquisition

Macular OCTA images (3 × 3-mm region) were acquired using an SS-OCTA device (PLEX Elite 9000, Carl Zeiss Meditec). We repeatedly acquired 4–9 raster scan sets for one eye of each subject. The score of signal strength was displayed for each captured image, and we used the images with scores more than 8/10 for averaging. Further, we excluded images with artifacts resulting from blinking or poor fixation. Choriocapillaris en face OCTA images were generated by selecting 8-μm-thick slabs starting from 29 μm below the automatically segmented RPE. We selected slabs in this region because a previous study showed that this region was a potential candidate for slabs containing the choriocapillaris [24]. Subfoveal choroidal thickness was defined as the distance between the RPE and chorioscleral interface, and was measured manually by using B-scan images of SS-OCTA.

### Multiple en face OCTA image averaging

Four to nine choriocapillaris en face images were registered before image averaging. As previously reported, the superficial capillary plexus en face images were initially registered, followed by the choriocapillaris images [22, 24]. Thereafter, the 4–9 choriocapillaris images were averaged to generated a single en face OCTA image of the choriocapillaris [24].

### Image analysis

**Fig 1:**
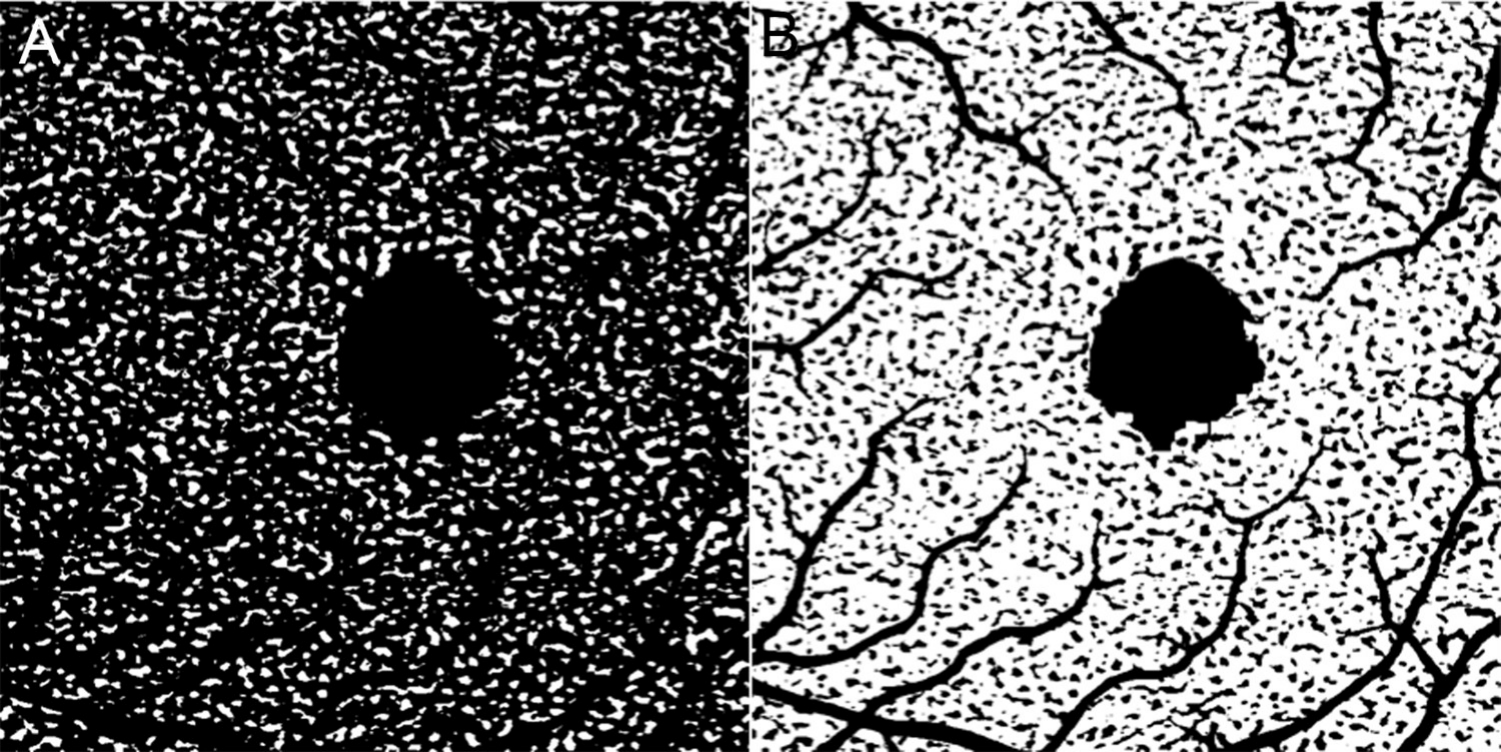
Binarized OCTA images of the choriocapillaris for quantitative image analysis. A. The image used for analyzing the flow voids without flow signals; the flow voids are white, and the vessels are black. B. The image used for analyzing the vessels; the vessels are white, and the flow voids are black. The image on the “left” is the inverted image. However, the major vessels and FAZ appear black as they are excluded from this analysis.

Binarization of the choriocapillaris images was conducted using the Phansalkar method, and the flow voids without flow signals and the vessels were quantitatively analyzed (Fig 1), as previously described [22, 24]. The number of flow voids and their total and individual areas were calculated using the binarized images. Moreover, the vessel diameter index (VDI) was calculated using inverted images [22, 24]. The VDI was defined as the average vessel caliber, which was calculated by dividing the total vessel area by the total vessel length. Regions containing major retinal vessels and the foveal avascular zone (FAZ) were excluded from the quantitative analyses [22, 24]. All image processing was automatically completed using ImageJ (available at http://rsb.info.nih.gov/ij/index.html).

### Statistical analysis

The differences between men and women were analyzed using unpaired *t* tests. We checked the distribution by Levene’s test before employing *t* test. The Pearson product moment correlation coefficient was used for analyzing the effects of age, axial length, and choroidal thickness. Partial correlation was used for adjusting for age. Statistical analyses were performed using SPSS for Windows/Macintosh, Version 2; SPSS Inc., Chicago, IL). P-values < 0.05 were considered statistically significant.

## Results

Although we initially recruited 88 subjects, 7 were excluded because of poor fixation or poor image quality. The characteristics of the included subjects are provided in Table 1.

**Table 1:** Characteristics of the study subjects.

Number of eyes	81
Age (years)	59.5 ± 16.2
Sex (male/female)	37(46%)/44(54%)
Axial length (mm)	24.26 ± 1.14
Subfoveal choroidal thickness (μm)	238.7 ± 56.8

**Fig 2:**
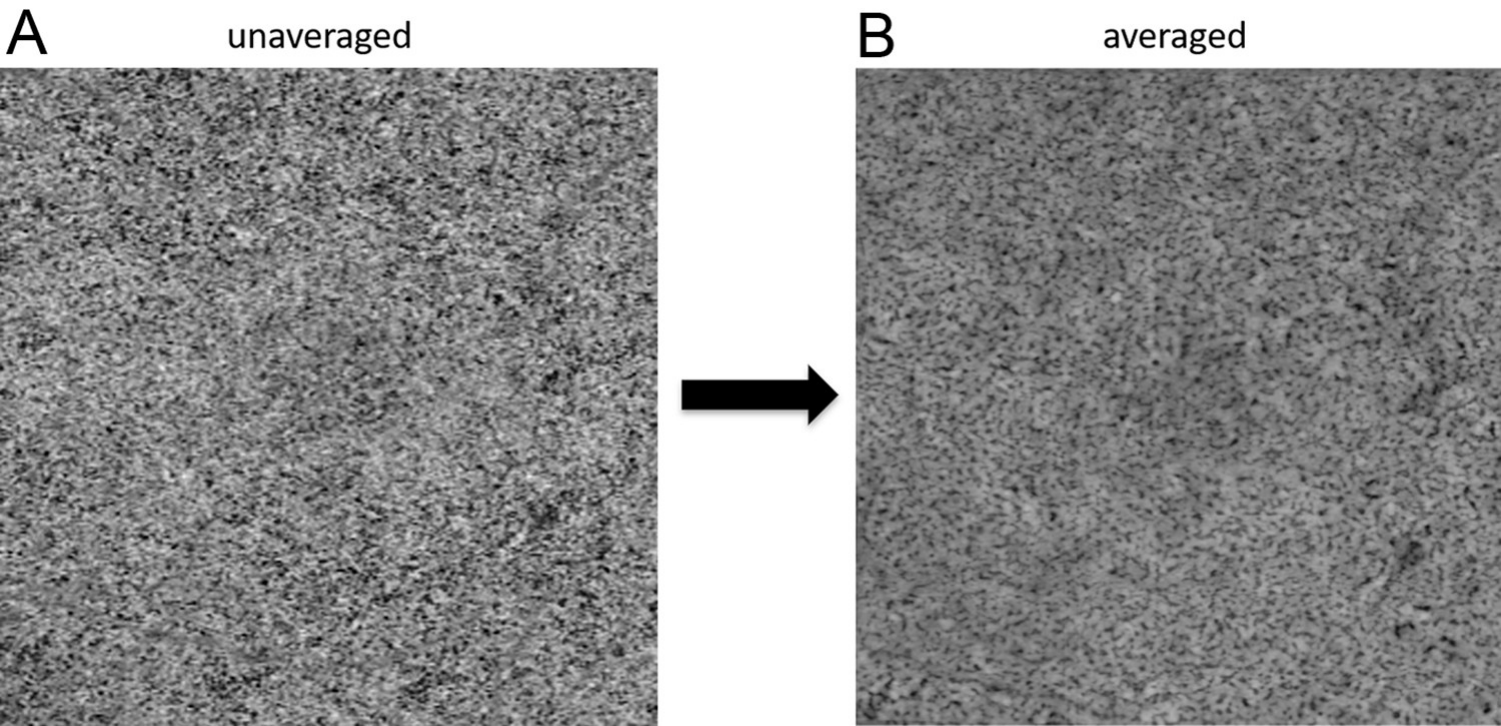
Averaged en face OCTA images of the choriocapillaris. A. A single en face OCTA image of the choriocapillaris before averaging. A granular appearance is observed. B. An en face OCTA image of the choriocapillaris after averaging. A continuous capillary meshwork is observed.

Bright areas on OCTA images are believed to correspond to blood flow, while the dark areas indicate flow voids [25]. Although the unaveraged choriocapillaris en face images had a granular appearance, the averaged images of all the eyes showed a continuous capillary meshwork, similar to the histology of the human choriocapillaris [1, 3] (Fig 2).

**Fig 3:**
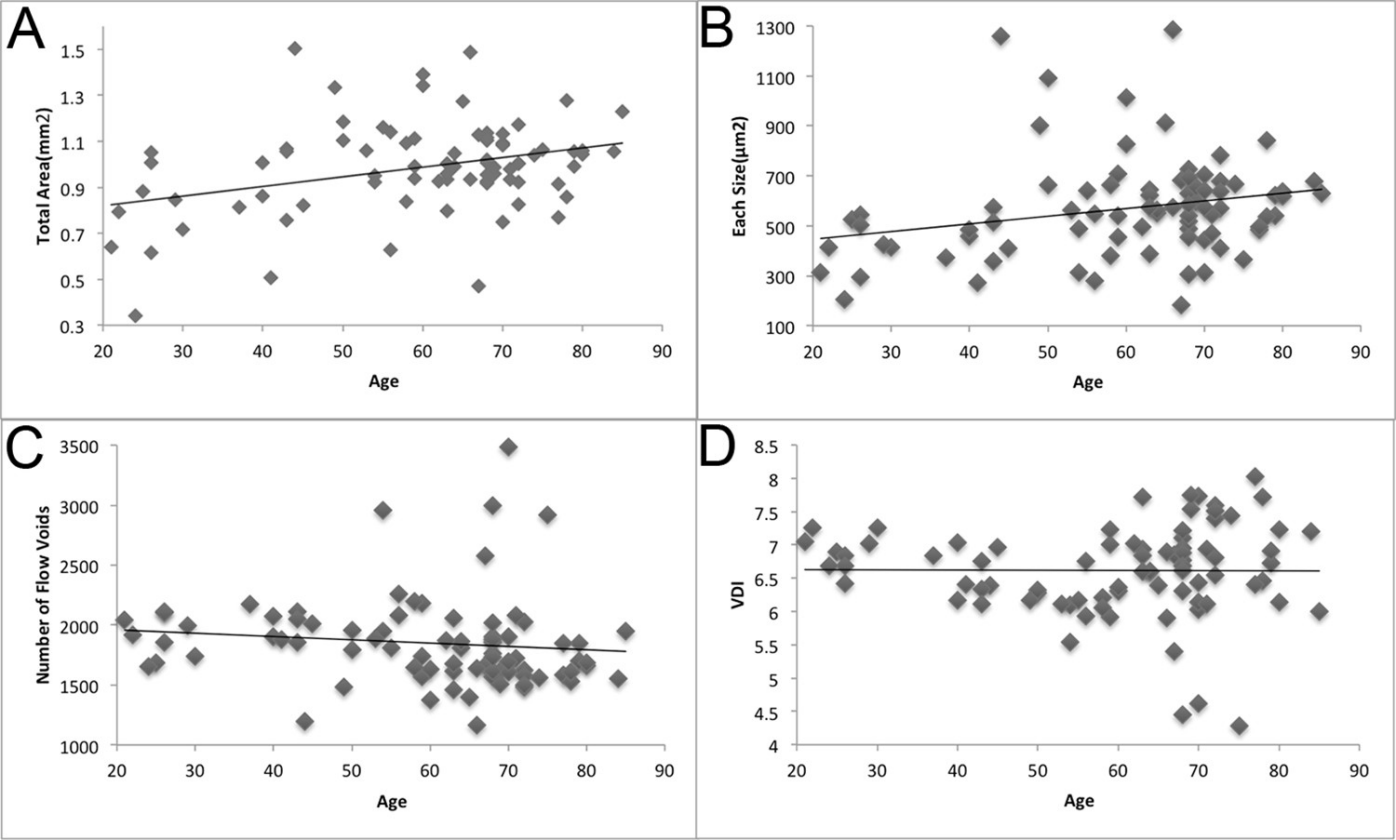
Correlations among age, total area, size, and number of flow voids, and the VDI. A. Correlation between the total area of flow voids and age. The mean total area is 0.99 ± 0.20 mm^2^. The total area positively correlates with age (p = 0.002, R = 0.336). B. Correlation between the size of flow voids and age. The mean size of flow voids is 567.8 ± 201.5 μm^2^. The size of flow voids positively correlates with age (p = 0.026, R = 0.247). C. Correlation between the number of flow voids and age. The mean number of flow voids is 1851 ± 375. No significant correlation is observed between the number of flow voids and age (p = 0.290). D. Correlation between the VDI of the flow voids and age. The mean VDI is 5.82 ± 0.49. No significant correlation is observed between the VDI and age (p = 0.947).

**Fig 4:**
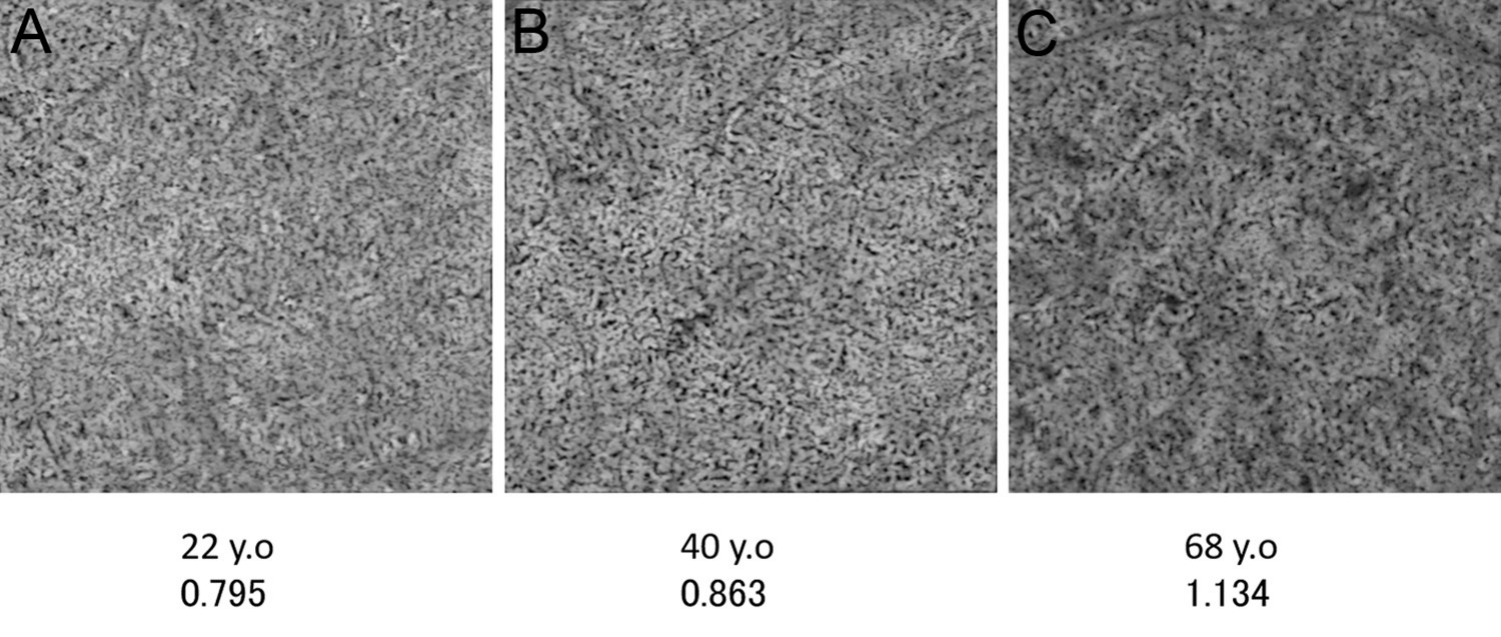
Representative en face OCTA images of the choriocapillaris in each age group. A. Image from a 22-year-old woman; the total area, size, and number of flow voids are 0.80 mm^2^, 415 μm^2^, and 1921, respectively. The VDI is 7.25. B. Image from a 40-year-old man; the total area, size, and number of flow voids are 0.86 mm^2^, 457 μm^2^, and 1899, respectively. The VDI is 7.04. C. Image from a 68-year-old man; the total area, size, and number of flow voids are 1.13 mm^2^, 727 μm^2^, and 1569, respectively. The VDI is 7.21.

The mean total area and size of flow voids were 0.99 ± 0.20 mm^2^ and 567.8 ± 201.5 μm^2^, respectively. They correlated positively with age (p = 0.002, R = 0.336 and p = 0.026, R = 0.247, respectively) (Fig 3). The mean total area and size of flow voids were 0.77 ± 0.20 mm^2^ and 401.6 ± 102.6 μm^2^, respectively, in the age group 20–39 years, 1.00 ± 0.21 mm^2^ and 571.3 ± 241.8 μm^2^, respectively, in the age group 40–59 years, and 1.02 ± 0.17 mm^2^ and 600.1 ± 179.0 μm^2^, respectively, in the age group ≥60 years. The age-related gains in the mean total area and size of flow voids were 4.20 × 10^−3^ mm^2^ and 3.07 μm^2^ per year, respectively. The mean number of flow voids was 1851 ± 375. No significant correlation was observed between the number of flow voids and age (p = 0.290) (Fig 3). The mean VDI was 5.82 ± 0.49. No significant correlation was observed between the VDI and age (p = 0.947) (Fig 3). Representative images from each age group are shown in Fig 4.

**Fig 5:**
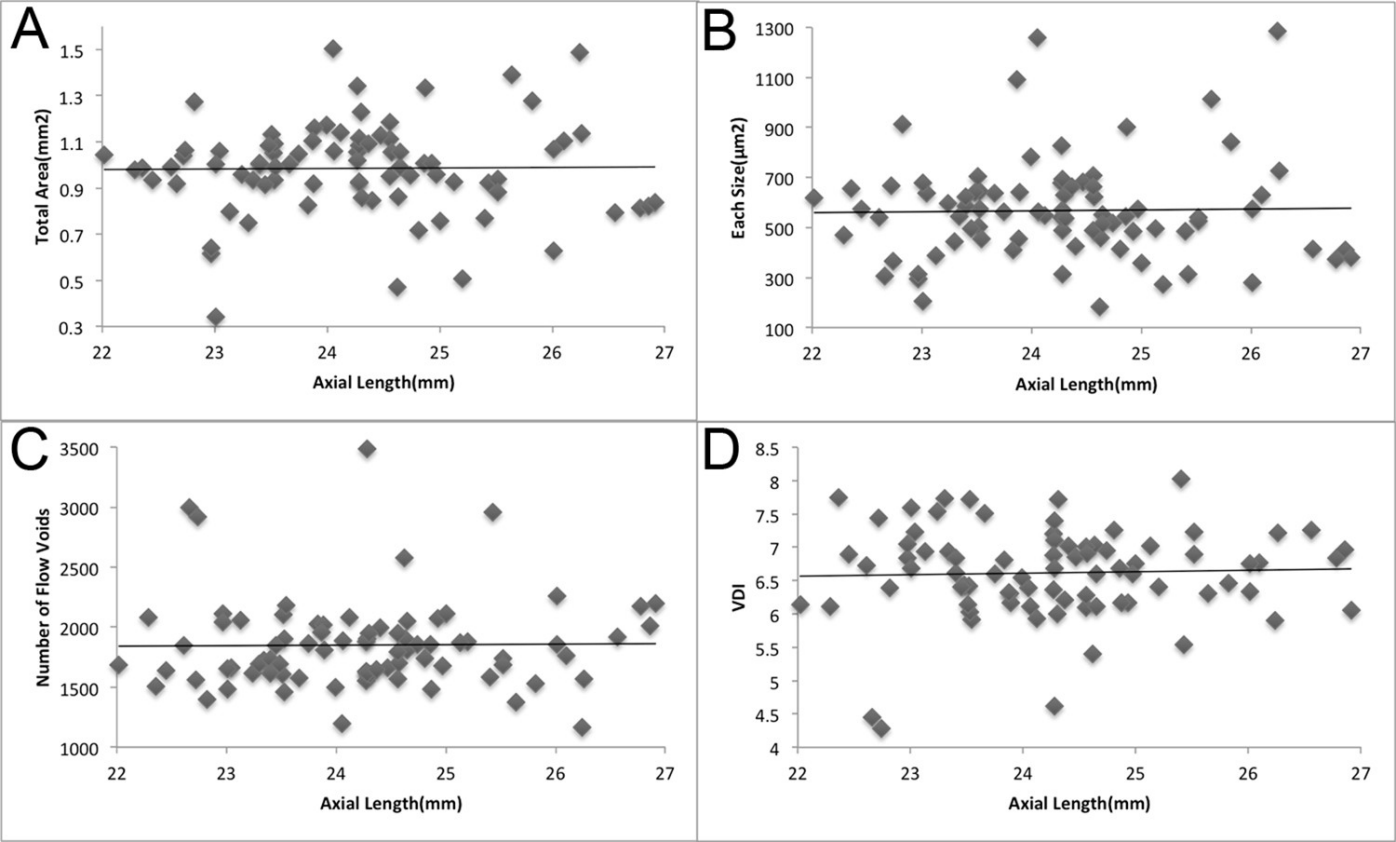
Correlations among axial length, total area, size, and number of flow voids, and the VDI. A. Correlation between the total area of flow voids and axial length. No significant correlation is observed between the total area of flow voids and axial length (p = 0.905). B. Correlation between the size of flow voids and axial length. No significant correlation is observed between the size of flow voids and axial length (p = 0.858). C. Correlation between the number of flow voids and axial length. No significant correlation is observed between the number of flow voids and axial length (p = 0.907). D. Correlation between the VDI and axial length. No significant correlation is observed between the VDI and axial length (p = 0.739).

**Fig 6:**
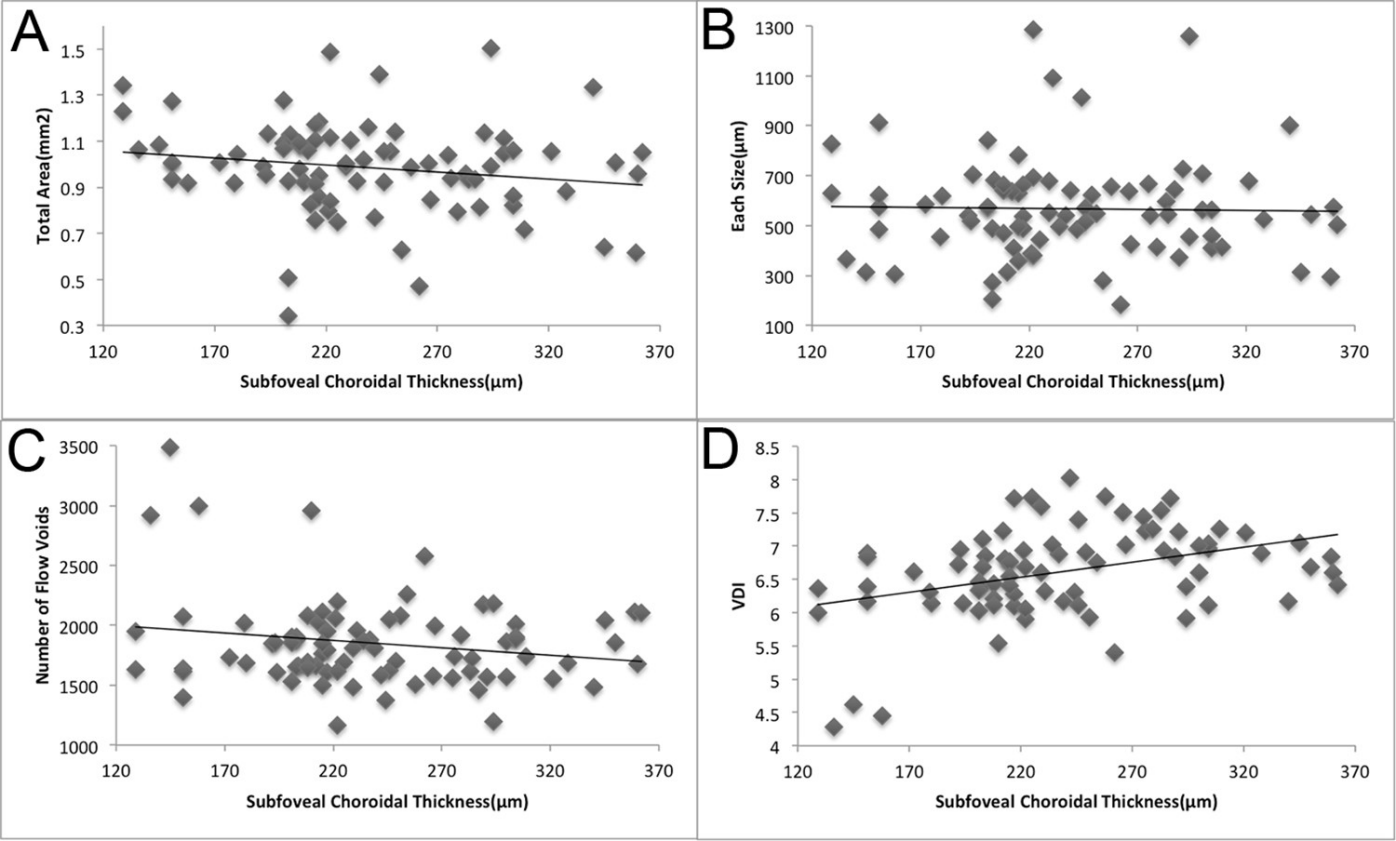
Correlations among subfoveal choroidal thickness, total area, size, and number of flow voids, and the VDI. A. Correlation between the total area of flow voids and subfoveal choroidal thickness. No significant correlation is observed between the total area of flow voids and subfoveal choroidal thickness (p = 0.125). B. Correlation between the size of flow voids and subfoveal choroidal thickness. No significant correlation is observed between the size of flow voids and subfoveal choroidal thickness (p = 0.838). C. Correlation between the number of flow voids and subfoveal choroidal thickness. No significant correlation is observed between the number of flow voids and subfoveal choroidal thickness (p = 0.093). D. Correlation between the VDI and subfoveal choroidal thickness. The VDI correlates positively with subfoveal choroidal thickness (p = 0.001, R = 0.377).

The total area, size, and number of flow voids, as well as the VDI did not correlate with axial length (p = 0.905, 0.858, 0.907, and 0.739, respectively) (Fig 5). However, the VDI correlated positively with subfoveal choroidal thickness (p = 0.001, R = 0.377) (Fig 6). The total area, size, and number of flow voids did not correlate with subfoveal choroidal thickness (p = 0.125, 0.838, and 0.093, respectively) (Fig 6).

After adjusting for age, the total area, size, and number of flow voids and the VDI did not correlate with axial length (p = 0.279, 0.383, 0.844, and 0.745, respectively). After adjusting for age, the total area and size of flow voids did not correlate with subfoveal choroidal thickness (p = 0.882 and 0.336, respectively). After adjusting for age, the number of flow voids correlated negatively and the VDI correlated positively with subfoveal choroidal thickness (p = 0.012 and <0.01, respectively).

However, no significant differences were observed in the mean total area, size, and number of flow voids, and the VDI between men and women after adjusting for age (p = 0.735, 0.994, 0.848, and 0.720, respectively) (Table 2).

**Table 2:** Correlations among axial length, subfoveal choroidal thickness, and sex after adjusting for age.

Variable	Total area of flow voids	Size of flow voids	Number of flow voids	VDI
Axial length	0.279	0.383	0.844	0.745
Subfoveal choroidal thickness	0.882	0.336	0.012*	<0.01*
Sex	0.735	0.994	0.848	0.720

*Statistically significant

All data are available in S1 File.

## Discussion

Previous studies analyzing normal choriocapillaris structure have used single OCTA images; however, the acquisition of accurate data on the choriocapillaris using single OCTA images is limited by its densely packed and interconnected arrangement with small intercapillary spaces. In this study, we could clearly visualize the choriocapillaris by using multiple averaged en face SS-OCTA, and our results showed that the area of flow voids increased with age in normal eyes.

SS-OCTA offers several advantages in terms of imaging the choriocapillaris. It has a longer wavelength and higher penetration than does SD-OCTA. In addition, its higher scan rate and invisible scan light help decrease errors such as motion artifacts, thereby leading to successful image acquisition. In fact, in this study, only seven eyes were excluded because of motion artifacts or poor image quality. Although multiple en face image averaging is time-consuming, it can be more practical to use SS-OCTA. Through the process of averaging en face SS-OCTA images of the choriocapillaris, the granular pattern observed in a single OCTA image can be transformed into a meshwork appearance, and the averaged images show more continuous vessels.

Several studies have reported a decrease in the density of the choriocapillaris with advancing age [16, 25, 26]. Ramrattan et al. showed that the capillary density of the choriocapillaris diminished in a linear fashion from approximately 0.75 in the first decade of life to approximately 0.40 in the tenth decade of life, by analyzing histological sections of normal eyes [26]. Using SD-OCTA, Spaide revealed that the flow of the choriocapillaris follows a power law distribution, and showed that age and hypertension affected the choriocapillaris [25]. Recently Zheng et al. used SS-OCTA and reported that choriocapillaris flow deficits increased with age across the central 5 mm of the macula, but the greatest increase was found in the central 1-mm region in normal eyes [16]. However, the unaveraged choriocapillaris en face images show a granular appearance, which makes accurate assessment of microvascular structures difficult. In the current study, we measured the total area, size, and number of flow voids as well as the VDI by using clearly visualized choriocapillaris images obtained via multiple enface SS-OCTA averaging. These results confirmed previous histological and OCTA studies, i.e., the total area and size of flow voids correlated positively with age in normal eyes.

In the current study, the VDI correlated positively with subfoveal choroidal thickness. This result confirmed the histological findings of Ramrattan et al. [26], who showed that choriocapillaris diameter also correlated with age to a much lesser extent. However, in the current study, the VDI did not correlate with age. Judging from these results, it seems that the choriocapillaris diameter correlated more strongly with choroidal thickness than with age.

Many studies revealed that axial length and sex as well as age affected retinal and choroidal thicknesses in normal eyes. For example, we previously reported that macular retinal thickness was greater in men than in women [27]. Moreover, inner retinal thickness decreased with increasing age [28]. Choroidal thickness was also greater in men than in women and negatively correlated with age and axial length [29]. Furthermore, retinal vascular density negatively correlated with age, but showed no difference between men and women [30]. In the current study, the total area, size, and number of flow voids as well as the VDI did not correlate with axial length, which was consistent with the results of the OCTA study by Zheng et al. [16]. The total area and size of flow voids did not correlate with subfoveal choroidal thickness even after adjusting for age, which was similar to the histological findings of Ramrattan et al. [26]. In addition, no significant differences were observed in the mean total area, size, and number of flow voids as well as the VDI between men and women. Taken together, these findings suggest that age, and not axial length, sex, or subfoveal choroidal thickness, is the most significant factor causing structural changes in the choriocapillaris in normal eyes. However, the current study excluded subjects with high myopia. A recent SD-OCTA study described decreased choriocapillaris flow in myopic eyes [21]. Therefore, further large-scale studies including patients with high myopia are necessary to investigate the effect of age, sex, axial length, and subfoveal choroidal thickness on the choriocapillaris.

This study has several limitations. First, the vessel caliber might be influenced by slight misalignment between the registered images. Second, to avoid the potential influence on quantitative analysis, regions of major retinal vessels and the FAZ were excluded. Third, this procedure involved some postprocessing using ImageJ.

Despite these limitations, the choriocapillaris structure was better visualized using multiple en face averaging of SS-OCTA images. The total area and size of flow voids within a 3 × 3-mm macular region positively correlated with age. These findings highlight the importance of understanding the changes that occur in the choriocapillaris with normal aging, as this would help investigate and identify the disease-specific changes in the choriocapillaris that arise in macular diseases.

## Supporting information

S1 File All data of this study. (XLSX)
